# Phosphoproteomic Analysis of Two Contrasting Maize Inbred Lines Provides Insights into the Mechanism of Salt-Stress Tolerance

**DOI:** 10.3390/ijms20081886

**Published:** 2019-04-16

**Authors:** Xiaoyun Zhao, Xue Bai, Caifu Jiang, Zhen Li

**Affiliations:** State Key Laboratory of Plant Physiology and Biochemistry, College of Biological Sciences, China Agricultural University, Beijing 100193, China; xiaoyunzhao@cau.edu.cn (X.Z.); xuebai0827@163.com (X.B.); cfjiang@cau.edu.cn (C.J.)

**Keywords:** maize, phosphoproteomics, salt tolerance, label-free quantification, root and shoot

## Abstract

Salinity is a major abiotic stress that limits maize yield and quality throughout the world. We investigated phosphoproteomics differences between a salt-tolerant inbred line (Zheng58) and a salt-sensitive inbred line (Chang7-2) in response to short-term salt stress using label-free quantitation. A total of 9448 unique phosphorylation sites from 4116 phosphoproteins in roots and shoots of Zheng58 and Chang7-2 were identified. A total of 209 and 243 differentially regulated phosphoproteins (DRPPs) in response to NaCl treatment were detected in roots and shoots, respectively. Functional analysis of these DRPPs showed that they were involved in carbon metabolism, glutathione metabolism, transport, and signal transduction. Among these phosphoproteins, the expression of 6-phosphogluconate dehydrogenase 2, pyruvate dehydrogenase, phosphoenolpyruvate carboxykinase, glutamate decarboxylase, glutamate synthase, l-gulonolactone oxidase-like, potassium channel AKT1, high-affinity potassium transporter, sodium/hydrogen exchanger, and calcium/proton exchanger CAX1-like protein were significantly regulated in roots, while phosphoenolpyruvate carboxylase 1, phosphoenolpyruvate carboxykinase, sodium/hydrogen exchanger, plasma membrane intrinsic protein 2, glutathione transferases, and abscisic acid-insensitive 5-like protein were significantly regulated in shoots. Zheng58 may activate carbon metabolism, glutathione and ascorbic acid metabolism, potassium and sodium transportation, and the accumulation of glutamate to enhance its salt tolerance. Our results help to elucidate the mechanisms of salt response in maize seedlings. They also provide a basis for further study of the mechanism underlying salt response and tolerance in maize and other crops.

## 1. Introduction

Salt stress is a major factor that limits plant growth and development throughout the world. A thorough understanding of salt tolerance and response mechanisms is essential for the planting and breeding of crops [1,2]. Increased salt concentrations in plant roots and shoots cause ion toxicity, hyperosmotic stress, and oxidative damage, impair metabolic processes, and decrease photosynthetic efficiency in crops [2,3]. One mechanism that alleviates salt stress involves removal of sodium (Na^+^) from the cytoplasm by transporting Na^+^ into the vacuole or out of the cell [4]. This transportation is executed by Na/H exchangers (antiporters) and induced by salt stress [5,6,7]. Salt stress leads to the production of reactive oxygen species (ROS) in mitochondria, chloroplasts, and peroxisomes in plants, which causes oxidative damage to proteins, DNA, and lipids [8]. Elimination of excessive ROS via the glutathione–ascorbate cycle and maintaining tolerable salt levels inside the plant cells through exportation or compartmentalization are generally accepted as two major strategies used by plants to survive salinity stress [9]. In addition, it is reported that plants can synthesize and accumulate small molecules, such as proline, betaine, soluble sugars, and amino acids, to protect themselves from salt stress [10,11,12].

Mass spectrometry (MS)-based proteomics have played an indispensable role in large-scale characterization of complex protein mixtures. It can identify thousands of proteins and their modifications in a single experiment [13]. It has been widely used in proteomic and phosphoproteomic analysis on Arabidopsis [14,15,16,17], maize [18,19,20,21], rice [22,23,24], soybean [25,26,27], and sorghum [28,29]. A comparative phosphoproteomic and proteomic analysis on roots of soybean seedlings identified 2692 phosphoproteins and 5509 phosphorylation sites. Eighty-nine differentially abundant proteins were discovered, and a novel salt tolerance pathway involving chalcone metabolism, which was mediated by the phosphorylation of MYB transcription factors, was proposed [30]. A proteomics-based approach has also been used to identify salt-responsive proteins in leaves of salt-resistant Chinese wheat cultivar. Fifty-two differentially abundant proteins were identified, including H^+^-ATPases, glutathione S-transferase, ferritin, and triosephosphateisomerase, which were found to be upregulated under salt stress [31]. A proteomic comparison between maize seedling roots from two different inbred lines under NaCl treatment for 2 days was reported [32]. Twenty-eight differentially abundant proteins were identified, such as 14-3-3 proteins, plasma membrane intrinsic proteins (PIP1 and PIP2), ribosomal protein S8, and 60S ribosomal protein L3-1. These proteins were mainly involved in signal processing, water conservation, protein synthesis, and abiotic stress-tolerance.

Protein phosphorylation plays critical roles in various biological processes, including cell growth, proliferation, metabolism, signal transduction, and apoptosis [33,34]. Many stress-related signaling pathways are involved in protein phosphorylation [35,36,37,38]. It is estimated that more than 30% of the proteins are phosphorylated at any given time in a living cell [39]. The SOS (Salt Overly Sensitive) pathway is a well-studied salt response signaling pathway in plants [38,40,41,42,43,44]. Salt stress elicits a calcium signal in the cytoplasm, and the calcium-binding protein encoded by SOS3 senses and binds to these Ca^2+^ to activate SOS2, a serine/threonine protein kinase. The SOS3–SOS2 complex activates SOS1 (plasma membrane Na^+^/H^+^ antiporter) or other transporters. The SOS signaling pathway is conserved in rice and maize [45,46]. Moreover, several protein-kinase- and protein-phosphatase-related signal pathways are also involved in salt response [47]. Therefore, identification and quantification of phosphorylated peptides under salt stress are highly desirable in understanding the salt resistance/tolerance mechanism in plants.

Maize (*Zea mays* L.) is a glycophyte plant that is hypersensitive to salt stress [48,49]. Salt stress not only results in weak seeding, a short radicle, and a low survival rate [50], but also impairs the photosynthesis system and leads to decreased stomatal conductance and an increased intercellular carbon dioxide concentration [2]. Therefore, characterizing the salt-tolerance mechanism is essential for increasing corn yield. Different maize inbred lines exhibit different salt tolerance characteristics, and comparing the proteomics and phosphoproteomics changes between salt-tolerant and salt-sensitive varieties under saline conditions is an effective means to explore the mechanisms responsible for the diversity in tolerance to salt stress. At present, studies on the phosphorylation of maize under salt stress are still very limited [51,52]. Most studies limit themselves to a few specific proteins, and most of these proteomic and phosphoproteomics studies focus on long-term saline conditions (e.g., over 48 h of NaCl treatment) [53,54]. Under salt stress, receptors located on the cell membrane quickly sense changes in NaCl content in the external environment, and second messengers, such as Ca^2+^, inositol phosphate, ROS, and phytohormones, are rapidly produced in the cytoplasm to transduce and amplify the salt stress signals, which, in turn, induce immediate phosphorylation of protein kinases and their downstream substrates [55,56]. Therefore, in-depth analysis of phosphoproteomics between salt-sensitive and salt-tolerant maize inbred lines under short-term salt treatment (0.5 to 2 h after salt treatment) is critical to obtain a comprehensive understanding of the salt response and salt-tolerance mechanism in maize.

In this study, an MS-based label-free quantitative phosphoproteomics analysis was employed to identify differentially regulated phosphoproteins in two maize cultivars: the salt-sensitive Chang7-2 and the salt-tolerant Zheng58. Here, 209 and 243 phosphoproteins were found to be significantly regulated in roots and shoots of the two maize lines after 100 mM NaCl treatment for 0.5 h and 2 h. Physiological parameters, such as hydrogen peroxide content, proline content, and metal ion contents, were examined in roots and shoots (Figure S1). The results of this study help to elucidate the salt response and regulation mechanism in maize and provide a theoretical basis for the cultivation of salt-tolerant varieties.

## 2. Results

### 2.1. Physiological Assays of Chang7-2 and Zheng58 in Response to Salt Stress

#### 2.1.1. Proline and H_2_O_2_ Contents under Salt Treatment

**Figure 1:**
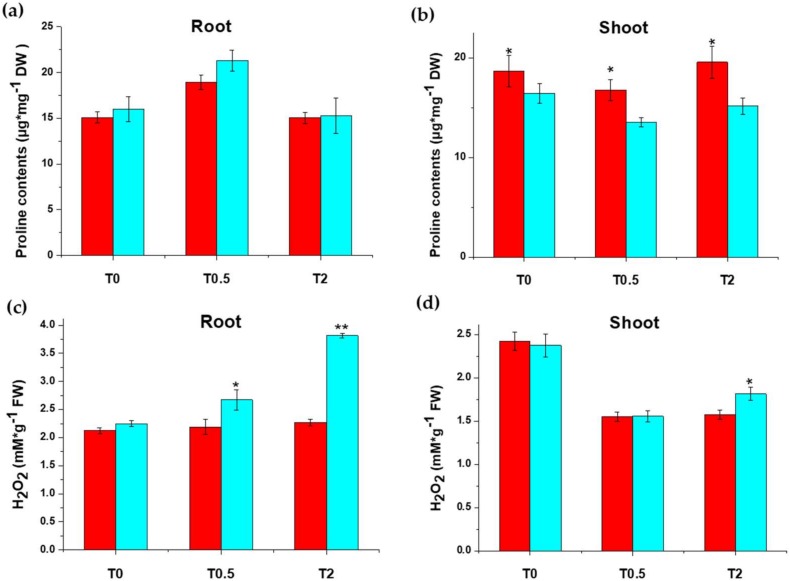
Physiological analysis of Chang7-2 and Zheng58 seedlings under saline conditions (100 mM NaCl treatment for 0.5 h and 2 h). Proline content of root (**a**) and shoot (**b**); Hydrogen peroxide content of root (**c**) and shoot (**d**). * indicates significant difference at 0.05; ** indicates a difference at the 0.01 level determined by Student’s *t*-test. Red, Chang7-2; Light green, Zheng58.

To investigate the difference in salt tolerance between Chang7-2 and Zheng58, we measured the hydrogen peroxide (H_2_O_2_) content, the proline content, and the metal ion contents (Na^+^, K^+^, and Mg^2+^ etc.) in the roots and shoots of the two cultivars. It can be seen that the proline content in roots increased significantly in both inbred lines after salt treatment for 0.5 h and the extent of increase was greater in Zheng58 (Figure 1a). The proline content dropped to the original level 2 h after treatment. The proline content in shoots showed the opposite trend, that is, the proline content in shoots decreased after 0.5 h of salt treatment and recovered after 2 h of treatment (Figure 1b). These results suggest that, under short-term salt stress, plants preferentially transport existing proline from shoots to roots instead of de novo synthesis [57,58]. Salt stress usually induces excessive amounts of H_2_O_2_ in plants. We found that the H_2_O_2_ content in roots of Zheng58 increased significantly after exposure to salt for 2 h but was unaffected in Chang7-2 (Figure 1c). The H_2_O_2_ content in shoots decreased significantly in both Chang7-2 and Zheng58 after exposure to salt for 0.5 h, and almost stayed the same at 2 h (Figure 1d). Thus, salt treatment may induce rapid accumulation of H_2_O_2_ in salt-tolerant plants to stimulate the ROS scavenging system.

#### 2.1.2. Metal Ions Response to Salt Stress in Chang7-2 and Zheng58

**Figure 2:**
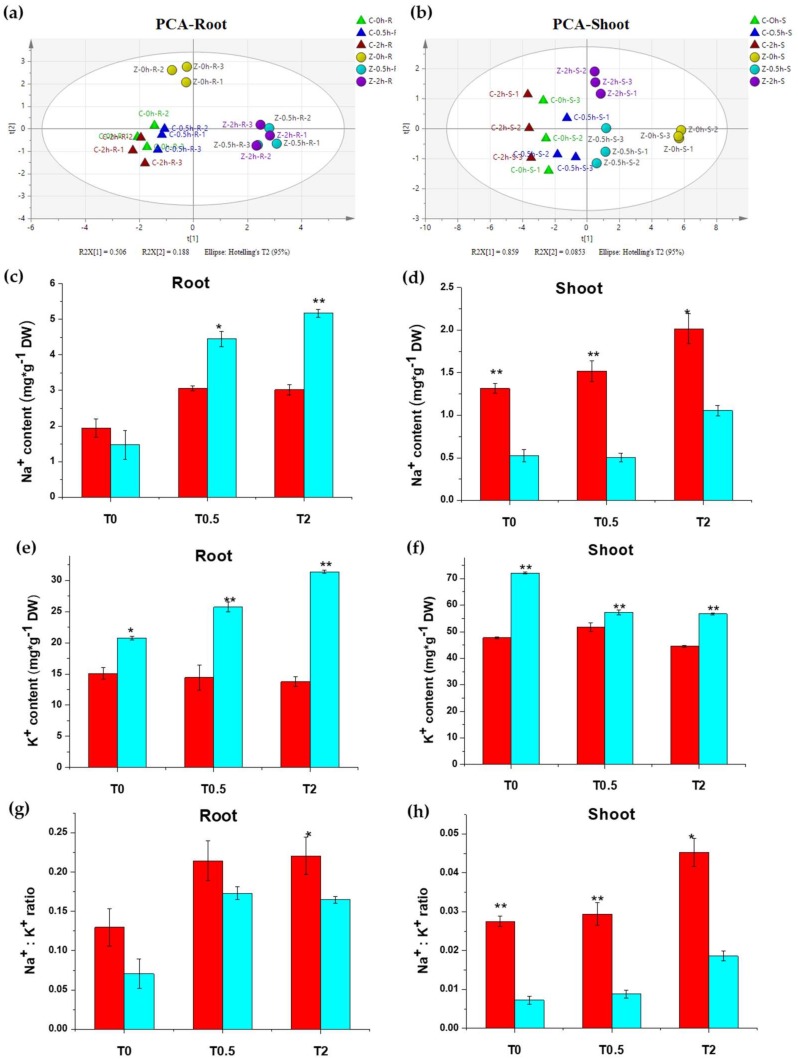
Metal ion contents in Chang7-2 and Zheng58 after salt treatment for 0.5 h and 2 h. Principal Component Analysis (PCA) score plot for root (**a**) and shoot (**b**); Na^+^ content (**c**,**d**); K^+^ content (**e**,**f**); and Na^+^/K^+^ ratio (**g**,**h**) for root and shoot. * indicates significant difference at 0.05; ** indicates a difference at the 0.01 level determined by Student’s *t*-test. Red, Chang7-2; Light green, Zheng58.

The fluctuation of metal elements in Chang7-2 and Zheng58 under salt treatment was measured by ICP-OES. K^+^ contents were significantly higher in shoots than in roots, while Mn^2+^ and Zn^2+^ contents were lower than in roots (Table S1). Principal component analysis (PCA) of all eight metal elements (K^+^, Na^+^, Ca^2+^, Mg^2+^, Mn^2+^, Fe^2+^, Zn^2+^, and Cu^2+^) in roots showed that there were distinct differences in ion contents between the two inbred lines, even before salt treatment. For Zheng58, the treatment groups at 0.5 h and 2 h can be clearly separated from the control group, but the difference between the two salt treatment groups was not significant. For Chang7-2, the control group and the treatment groups could not be completely separated on the PCA score plot (Figure 2a), suggesting that salt stress influences the ion content of Zheng58 to a higher extent than that of Chang7-2. K^+^, Ca^2+^, and Fe^2+^ had the highest loading in PC1, while Mg^2+^ and Mn^2+^ contributed most to PC2 (Figure S2a). Similar to the results from roots, salt treatment induced significant changes on metal ion contents in shoots of Zheng58 but not in Chang7-2 (Figure 2b). K^+^, Na^+^, and Ca^2+^ were the key elements responsible for such differences (Figure S2b).

Ionomic data showed that the K^+^ and Na^+^ content in both roots and shoots changed significantly after salt treatment. One interesting finding was that Na^+^ contents in the roots of Zheng58 were much higher than those of Chang7-2 after salt treatment, while in shoots, it was Chang7-2 that had the higher Na^+^ content (Figure 2c,d). Such a finding indicates that, under salt stress, Zheng58 greatly reduced Na^+^ transportation from roots to shoots, which is an effective strategy for salt-tolerant plants to resist salt stress [59,60]. Also, the K^+^ content increased significantly in the roots of Zheng58 but was unaffected in Chang7-2 under salt treatment (Figure 2e), and the Na^+^/K^+^ ratio was much lower in Zheng58 than Chang7-2 in both roots and shoots (Figure 2g,h). Taken together, when faced with salt stress, Zheng58 maintained a lower Na^+^ concentration and Na^+^/K^+^ ratio in shoots to remain viable [60].

### 2.2. Overview of Phosphoproteins Identified in Maize Seedlings

**Figure 3:**
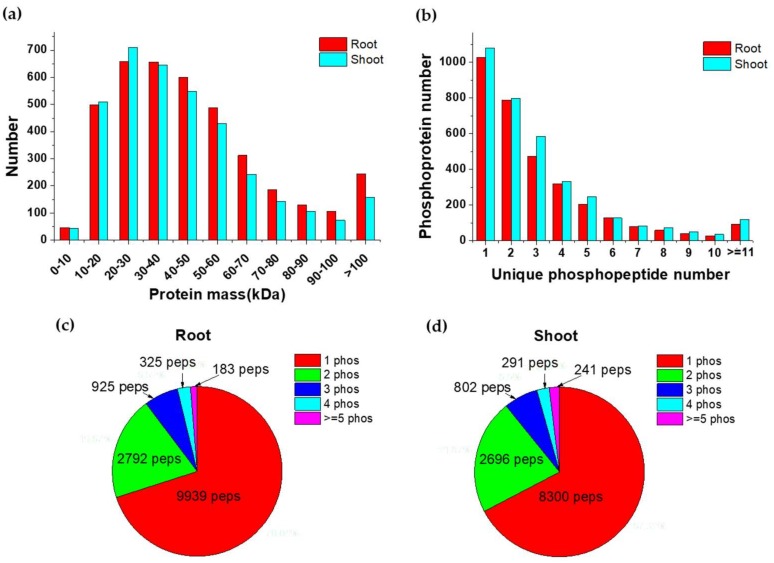
Overview of phosphoprotein identification. Distribution of protein mass (**a**) and numbers of unique peptides (**b**) of identified proteins. The phosphorylation status of the identified phosphopeptides in roots (**c**) and shoots (**d**).

Label-free quantitation of phosphoproteins after salt treatment was realized with Progenesis QI for Proteomics and protein identification was realized by the Mascot search engine. In roots and shoots, 36,635 and 40,119 peptide spectrum matches (PSMs) were identified separately at a 1% false discovery rate (FDR). In roots, 9772 unique phosphopeptides assigning to 3084 phosphoproteins were quantified and 11,062 unique phosphopeptides mapping to 2986 phosphoproteins were quantified in shoots. Altogether, 9448 unique phosphorylation sites from 4116 phosphoproteins were detected, and more than 7000 phosphorylation sites can be detected from each sample (Table S2). We detected 2211 and 2454 proteins in roots and shoots, respectively, with at least two unique phosphopeptides, accounting for more than one half of the identified phosphoproteins (Figure 3a). Most of the identified peptides contained one phosphorylation site, and 4225 and 4030 peptides in roots and shoots, respectively, were identified with multiple phosphorylation sites (Figure 3c,d).

### 2.3. Differentially Regulated Phosphoproteins in Chang7-2 and Zheng58 in Response to Salt Treatment

**Figure 4:**
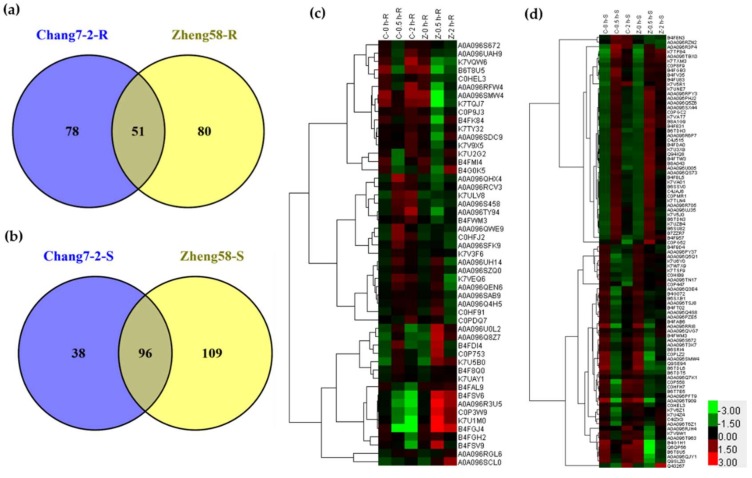
Differently regulated phosphoproteins (DRPPs) in salt-treated Chang7-2 and Zheng58 in roots (**a**) and shoots (**b**); clustering analysis of DRPPs detected in both Chang7-2 and Zheng58 seedlings ((**c**), roots and (**d**), shoots).

Differentially regulated phosphoproteins were defined as phosphoproteins with more than 2-fold of the changes in intensity after 0.5 h or 2 h of salt treatment when compared with their respective control group (*p* < 0.05). For the roots, 129 phosphoproteins (99 increased and 30 decreased) from Chang7-2 and 131 phosphoproteins (42 increased and 89 decreased) from Zheng58 met the above criteria (Figure 4a and Figure S3a,b). Fifty-one phosphoproteins were identified in both lines, and 78 and 80 phosphoproteins were unique to Chang7-2 and Zheng58, respectively. (Figure 4a). For the shoots, 134 phosphoproteins (74 upregulated and 60 downregulated) from Chang7-2 and 205 phosphoproteins (136 upregulated and 69 downregulated) from Zheng58 were significantly changed after salt treatment (Figure 4b and Figure S3c,d). Among them, 96 phosphoproteins were shared between Chang7-2 and Zheng58 shoots, and 38 and 109 phosphoproteins were unique to Chang7-2 and Zheng58, respectively. (Figure 4b).

Of the 51 differentially regulated phosphoproteins (DRPPs) detected in the roots of both Chang7-2 and Zheng58, hierarchical clustering analysis showed that they exhibited different abundance patterns in different inbred lines (Figure 4c), indicating different response mechanisms of these two maize lines under salt stress. Among the 51 DRPPs, 29 DRPPs were upregulated in Chang7-2 but downregulated in Zheng58. These phosphoproteins included kinases, transcription factors, and ion-transport-related phosphoproteins. Five DRPPs were decreased in Chang7-2 but increased in Zheng58, which consisted of the 6-phosphogluconate dehydrogenase family protein, ribosomal protein, pyruvate dehydrogenase complex E1 alpha subunit, phosphoenolpyruvate carboxykinase, and putative l-gulonolactone oxidase. The remaining 17 DRPPs showed the same trend in the two lines. These 96 DRPPs in shoots shared between the two lines are clustered in Figure 4d. The trends of phosphorylation of these proteins in Chang 7-2 and Zheng 58 were also diverse.

### 2.4. Clustering Analysis of DRPPs Unique to Chang7-2 or Zheng58 in Maize Roots

**Figure 5:**
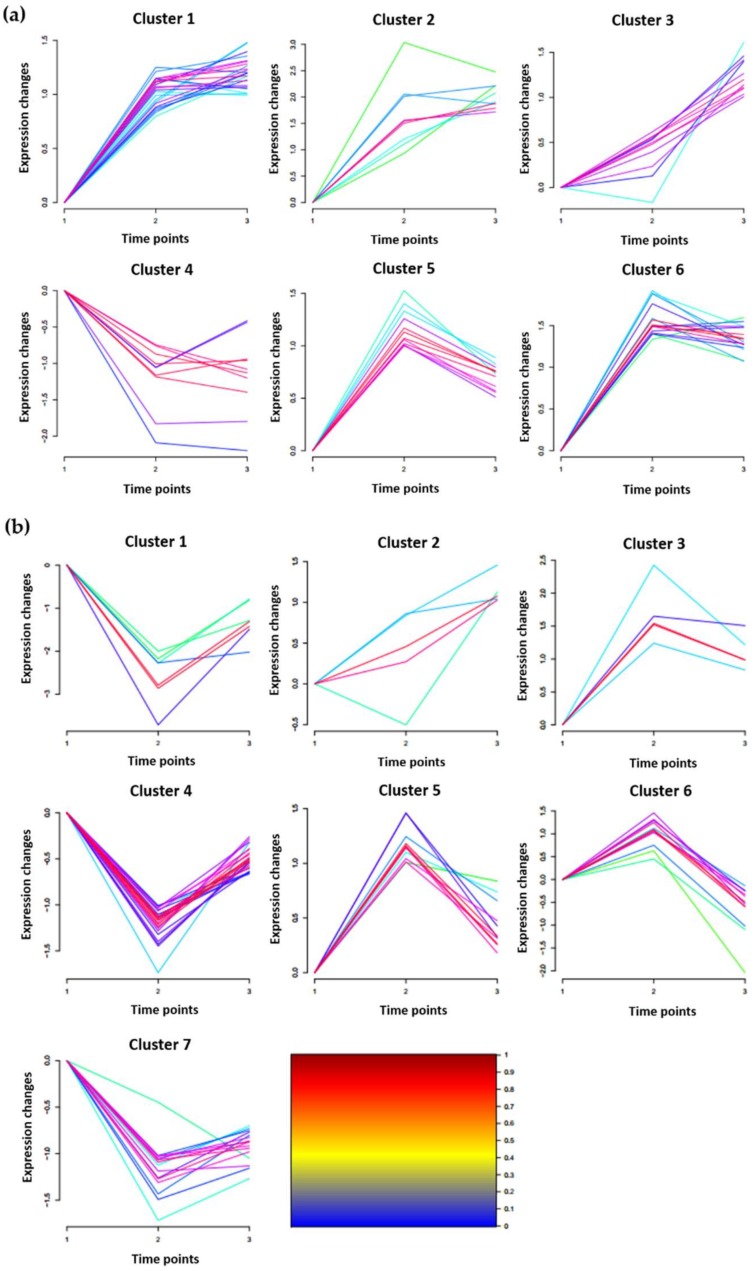
MFUZZ clustering analysis of DRPPs unique to Chang7-2 (**a**) and Zheng58 (**b**).

Clustering analysis of DRPPs unique to Chang7-2 or Zheng58 were conducted using the Mfuzz algorithm embedded in the Wukong data analysis platform [61]. DRPPs from Chang7-2 can be grouped into six categories (Figure 5A). The phosphorylation level of proteins in cluster 1 was significantly upregulated after 2 h of salt treatment. There were 23 phosphoproteins in this cluster. They were involved in metal ion transport, phosphatidylinositol phosphorylation, signal transduction, transcription, regulation of lipid kinase activity, and the cellulose biosynthetic process. Similar to cluster 1, DRPPs in cluster 2 were upregulated in response to salt stress, but the extent of the increase was greater than that in cluster 1. Proteins in this cluster included: chloride channel, putative casein kinase protein, and proteasomal ubiquitin receptor ADRM1-like. DRPPs that functioned in the cell surface receptor signaling pathway and intracellular protein transport were only significantly upregulated after 2 h treatment, and were assigned to cluster 3. Cluster 4 contained proteins that were downregulated after exposure to salt stress. DRPPs in cluster 5 and 6 were induced after 0.5 h of treatment, and recovered after 2 h. Proteins that played roles in protein transport, response to oxidative stress, and retrograde-vesicle-mediated transport were assigned to cluster 5, and proteins involved in signal transduction, the lipid metabolic process, and defense response to fungus were assigned to cluster 6 (Figure 5a).

Eighty DRPPs that were unique to Zheng58 roots after salt stress were clustered into seven categories (Figure 5b). DRPPs in cluster 1, 4, and 7 were all significantly downregulated at 0.5 h after salt treatment and recovered at 2 h. Proteins in cluster 1 decreased to a higher extent than those in clusters 4 and 7, and seven proteins, including plasma membrane ATPase (K7TX67) and UBP1-associated protein (C0P558), were in this cluster. Twenty-three DRPPs were in cluster 4. They were involved in such biological processes as cell redox homeostasis, transport, pentose-phosphate shunt, microtubule cytoskeleton organization, the glutamate metabolic process, rRNA processing, the auxin-activated signaling pathway, and regulation of transcription. The DRPPs in cluster 7 were proteins related to transcription initiation, nucleotide binding, and calcium ion binding. The DRPPs in clusters 3 and 5 were both upregulated at 0.5 h and then declined at 2 h. Serine/threonine-protein kinase SRK2A, mitogen-activated protein kinase, and ribosomal proteins were included in cluster 3. Potassium channel 5, glutamate synthase, uridine-cytidine kinase C-like, and several uncharacterized proteins were included in cluster 5. Twelve DPRRs that increased in abundance at 0.5 h and declined at 2 h were grouped into cluster 6, including sodium/hydrogen exchanger 2-like (A0A096Q7K1), high-affinity potassium transporter (W5U5W2), molybdate transporter 2-like (A0A096TR23), and protein NRT1/PTR family 8.3-like (A0A096TGV7).

### 2.5. Gene Ontology Analysis of Salt-Responsive DRPPs in the Two Inbred Lines

**Figure 6:**
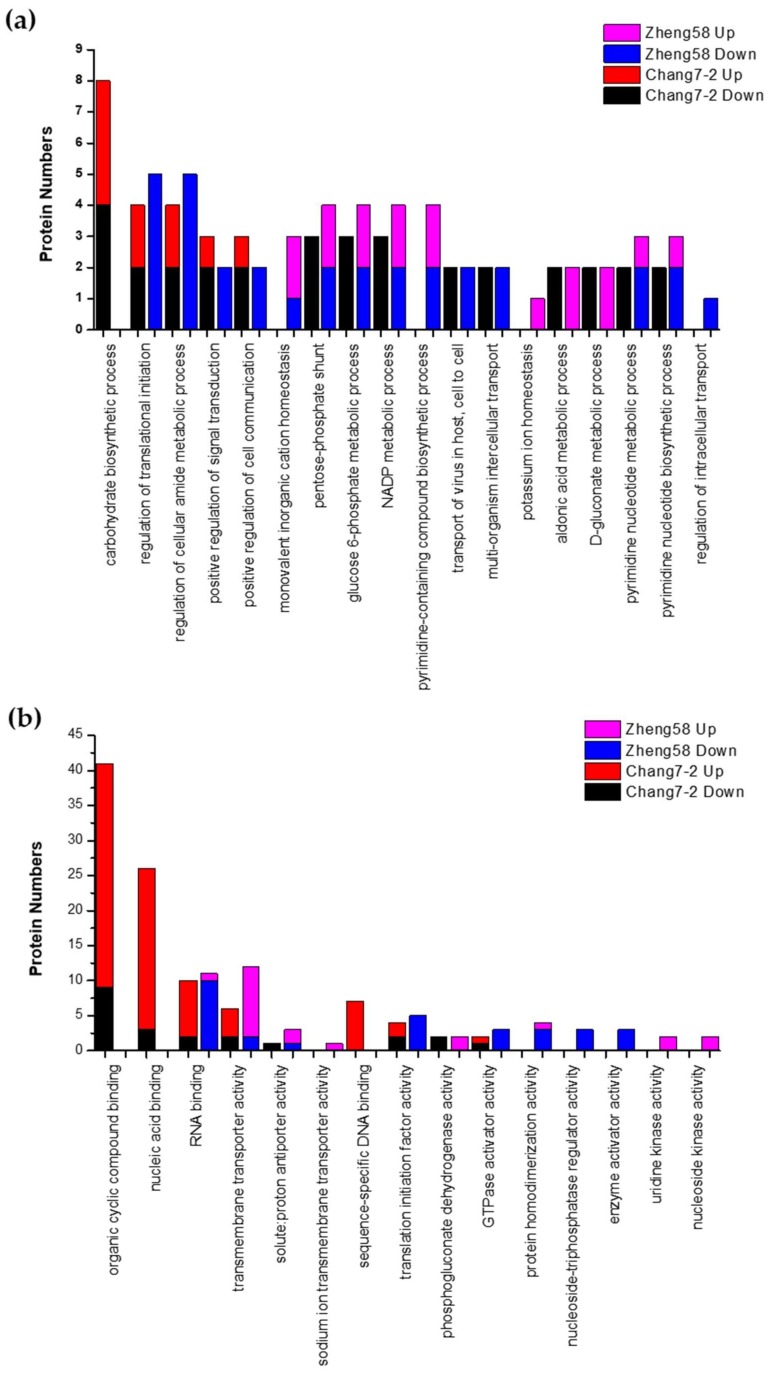
Gene Ontology (GO) analysis of DRPPs in roots of Chang7-2 and Zheng58. GO terms in biological processes (**a**) and molecular functions (**b**) are shown.

Gene ontology (GO) enrichment of salt-responsive DRPPs in Chang7-2 and Zheng58 roots were performed using the Wukong platform [61]. GO functional analysis revealed that most of the differentially phosphorylated proteins under salt stress in the two lines were involved in the same biological processes or were endowed with the same molecular functions, although some unique GO terms were also enriched for each individual line. As shown in Figure 6, 14 GO terms and 17 GO terms were enriched in Chang7-2 and Zheng58, respectively, in the biological process category. One GO term, carbohydrate biosynthetic process (GO: 0016051), was unique to Chang7-2. Four GO terms, including monovalent inorganic cation homeostasis (GO: 0055067), pyrimidine-containing compound biosynthetic process (GO: 0072528), potassium ion homeostasis (GO: 0055075), and regulation of intracellular transport (GO: 0032386), were unique in Zheng58. Among these, potassium ion homeostasis was upregulated and regulation of intracellular transport was downregulated in Zheng58. Moreover, two GO terms, aldonic acid metabolic process and D-gluconate metabolic process, were upregulated in Zheng58 but downregulated in Chang7-2. For the molecular function category, nine GO terms and 12 GO terms were enriched in Chang7-2 and Zheng58, respectively. Three GO terms were unique to Chang7-2, namely, organic cyclic compound binding (GO: 0097159), nucleic acid binding (GO: 0003676), and sequence-specific DNA binding (GO: 0043565). Six GO terms, including sodium ion transmembrane transporter activity (GO:0015081), protein homodimerization activity (GO: 0042803), nucleoside-triphosphatase regulator activity (GO: 0060589), enzyme activator activity (GO: 0008047), uridine kinase activity (GO: 0004849), and nucleoside kinase activity (GO: 0019206), were specifically enriched in Zheng58. Among these, sodium ion transmembrane transporter activity, uridine kinase activity, and nucleoside kinase activity were upregulated in Zheng58, and nucleoside-triphosphatase regulator activity and enzyme activator activity were downregulated in Zheng58 (Figure 6).

### 2.6. Protein–Protein Interaction Analysis

**Figure 7:**
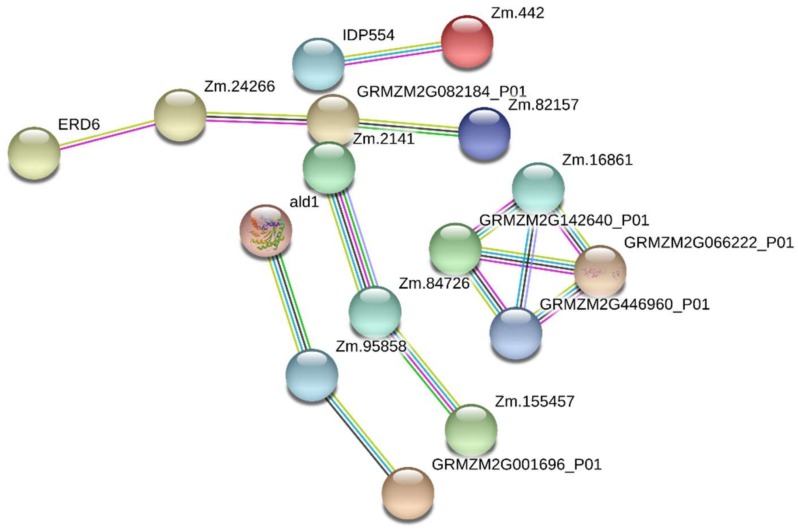
The protein interaction network of DRPPs in Zheng58 roots.

To investigate the transduction of salt stress signals in maize root cells, DRPPs identified in Chang7-2 and Zheng58 roots were further analyzed using the String 10.5 database (https://string-db.org/) [62]. Protein–protein interactions with confidence scores higher than 0.7 were shown. Five groups of interactions were significantly enriched in Zheng58 (Figure 7). Proteins including sodium/hydrogen exchanger (A0A096Q7K1, Zm.82157), sugar transport protein (C0P753, ERD6), glutamate synthase (A0A096RAH5, Zm.24266), and one uncharacterized protein (transport activity, B4FC34, GRMZM2G082184) constituted the first group. These proteins are related to sodium transport, amino acid transport/metabolism, and sugar transport. The second group was comprised of fructose-bisphosphate aldolase (B4FAL9, ALD1), pyruvate dehydrogenase E1 component subunit alpha (B4FGJ4, Zm.95858), and phosphoenolpyruvate carboxylase (C0P3W9, GRMZM2G001696). Proteins in this group are involved in carbon metabolism. In the third interaction group, uridine kinase (K7UTD0, Zm.84726) interacted with uridine-cytidine kinase C-like (A0A096SG98, Zm.2141) and shaggy-related protein kinase alpha-like (A0A096S672, Zm.155457). Proteins in this group are related to pyrimidine metabolism. The fourth group contained proteins involved in protein translation, including ribosomal protein S10 (J7LC26, Zm.16861), 40S ribosomal protein S27 (Q9ZQX9, GRMZM2G066222), 40S ribosomal protein S10-1 (B4FW06, GRMZM2G446960), and 60S ribosomal protein L24 (A0A096R3U5, GRMZM2G142640). Finally, calcium-binding protein (C0P445, IDP554), which is involved in plant–pathogen interaction, interacted with myosin1 (A0A096PFT4, Zm.442). They are connected with host defenses against pathogens. Additionally, five protein interaction groups were predicted for Chang7-2 roots as shown in Figure S4.

## 3. Discussion

**Figure 8:**
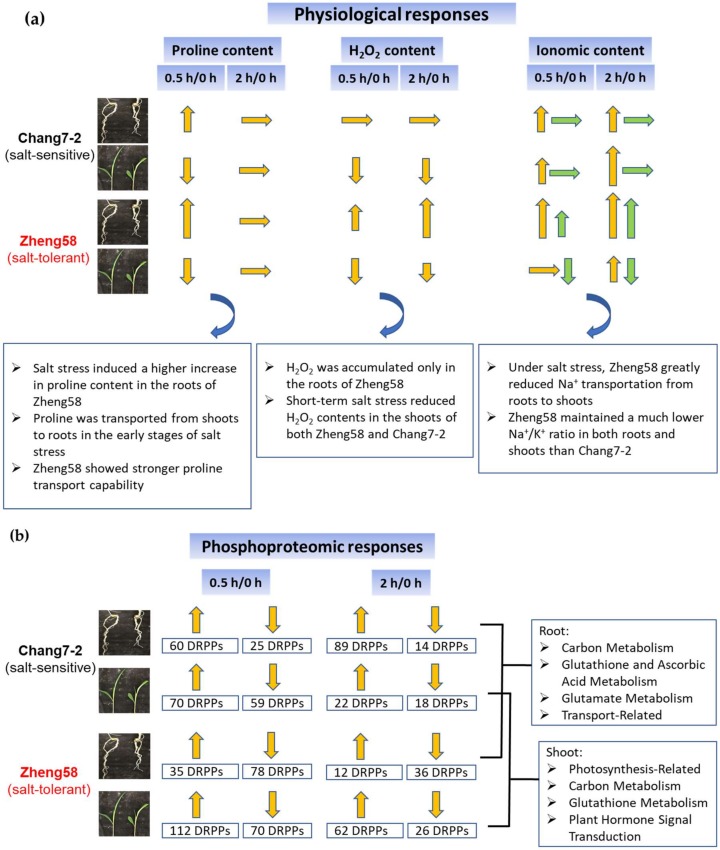
A summary of differences in physiology (**a**) and phosphoproteomics (**b**) in response to salinity stress in roots and shoots of Chang7-2 and Zheng58. The up arrows indicate upregulation and the down arrows indicate downregulation; The lengths of the arrows indicate the degree of changes; In the ionomic content section, the yellow arrows represent Na^+^ and the green arrows indicate K^+^.

Saline soil is a major threat to maize cultivation and yield enhancement. Therefore, characterizing the mechanism of salt tolerance in different maize varieties is critical for maize breeding and production. In this study, we compared the phosphoproteomics of two maize inbred lines with distinct salt tolerances in response to short-term salt treatment to explore the salt tolerance mechanism of maize. Most of the previous studies focus on proteomics and phosphoproteomics profiling under long-term salt treatment, i.e., proteomics studies were performed several days after salt treatment [54]. It is well-known that salt stress induces many processes related to protein phosphorylation in plants, which occurs in the time scale of seconds to minutes [38,47]. Therefore, to complement the salt-tolerant mechanism in maize, we investigated the short-term phosphoproteomics response of maize under salt stress conditions. We compared the phosphorylation levels of two contrasting inbred lines, one salt-tolerant (Zheng58) and one salt-sensitive (Chang7-2), in response to short-term (0.5 and 2 h) salt stress. Physiological changes in line with the phosphorylation events were also assessed to explore the salt tolerance mechanism in maize. Roots and shoots of seedlings from the two inbred lines were collected and assayed separately. A summary of the differences in physiology and phosphoproteomics in response to salinity stress in roots and shoots of Chang7-2 and Zheng58 is listed in Figure 8.

### 3.1. Physiological Difference between Zheng58 and Chang7-2 under Salt Stress

The physiological responses of Chang7-2 and Zheng58 to short-term salt treatment were evaluated to investigate their distinct salt endurance characteristics. In the roots of the two inbred lines, the proline content increased at 0.5 h of salt treatment and returned to the original levels at 2 h; the opposite trends were observed in the shoots (Figure 1a,b). Salt stress induced a larger increase in proline content in Zheng58, suggesting that Zheng58 mobilized the defense mechanism to cope with salt stress more quickly. The proline content of both inbred lines decreased to normal levels after 2 h of treatment, indicating that other defense strategies were initiated by the plants to adapt to the osmotic stress caused by salt stress after a certain period of salt treatment. In shoots, the proline content decreased at 0.5 h of salt treatment, and slightly rebounded at 2 h, which was the exact opposite of the change in proline content in roots. Thus, we proposed that the proline accumulated in roots was mainly transported from the shoots to initiate a quick response in the early stages of salt stress. Zheng58 showed a stronger proline transport capability; supporting it was its salt-tolerant nature.

The H_2_O_2_ contents in shoots were higher than those in roots for both maize lines under control conditions. Short-term salt stress induced H_2_O_2_ accumulation only in the roots of Zheng58. In the meanwhile, decreased H_2_O_2_ contents were observed in the shoots of both Zheng58 and Chang7-2 after salt treatment (Figure 1c,d). These results indicate that the roots of the salt-tolerant plant Zheng58 accumulated more H_2_O_2_ under short-term salt stress, and it had a higher tolerance to elevated H_2_O_2_. The H_2_O_2_ content decreased in shoots in both inbred lines after salt treatment, indicating immediate removal of H_2_O_2_ in the shoots.

Metal ions assay of the two inbred lines revealed that the metal ions contents in the two inbred lines were statistically different even before salt treatment, as indicated by principal component analysis (Figure 2a,b). The metal ions contents in the two lines showed different responses to salt stress. The metal ions contents in Zheng58 changed significantly after salt treatment, while the metal ions contents in Chang7-2 did not show notable changes under saline conditions. Zheng58 greatly reduced the transportation of Na^+^ from roots to shoots and maintained a much lower Na^+^/K^+^ ratio than Chang7-2 under salt stress (Figure 2g,h), demonstrating that Zheng58 had a better salt tolerance capability than Chang7-2.

### 3.2. Functional Analysis of DRPPs in Roots of Zheng58 and Chang7-2 under Salt Stress

#### 3.2.1. Carbon Metabolism

6-phosphogluconate dehydrogenase 2 (B4FSV6) catalyzes the dehydrogenation of 6-phosphate-gluconate to form 5-phosphate-ribulose; it is the key enzyme in the pentose phosphate pathway [63]. This reaction is accompanied by the production of NADPH, which is a reducing agent for various synthetic reactions in living systems. Moreover, NADPH is the cofactor required for oxidized glutathione to be converted to reduced glutathione. Glutathione is vital for redox homeostasis and is one of the main antioxidants involved in eliminating reactive oxygen species (ROS) [64]. The phosphorylation of 6-phosphogluconate dehydrogenase 2 was downregulated in Chang7-2 (0.37-fold and 0.31-fold at 0.5 and 2 h treatment, respectively, Table 1) but upregulated in Zheng58 (2.05-fold at 2 h treatment), suggesting that Zheng58 can produce more NADPH under salt stress. It has been reported that 6-phosphogluconate dehydrogenase was upregulated in salt-treated Jing724, a salt-tolerant maize line, but not in the salt-sensitive maize line D9H, consistent with our results [54].

B4FGJ4 (pyruvate dehydrogenase), B4FAL9 (aldolase 1), and C0P3W9 (phosphoenolpyruvate carboxykinase) are all involved in carbon metabolism. Pyruvate dehydrogenase catalyzes the dehydrogenation and decarboxylation of pyruvate to form acetyl-CoA, which is the core reaction of the Tricarboxylic acid (TCA) cycle [65]. Phosphoenolpyruvate carboxykinase functions as the catalytic enzyme in converting oxaloacetate to phosphoenolpyruvate, an intermediate product in glycolysis [65]. The increased phosphorylation of these two proteins (a 2.16 to 3.60-fold increase) in response to salt stress in Zheng58 helped the plant resist salt stress by producing more energy.

#### 3.2.2. Glutathione and Ascorbic Acid Metabolism

Glutathione S-transferase 3 (B4FK84) is the key enzyme that catalyzes the binding reaction between glutathione and various electrophilic compounds [66]. The phosphorylation level of this protein decreased at 0.5 h of salt treatment and then returned afterwards at 2 h in Chang7-2, but remained at a lower level in Zheng58 (Table 1). The lower phosphorylation level of glutathione S-transferase 3 was beneficial to the accumulation of glutathione, which is an important antioxidant to scavenge ROS and protect sulfhydryl groups in many proteins and enzymes [64]. Thus, the reduced phosphorylation of glutathione S-transferase 3 in Zheng58 suggests that the plant may maintain a higher level of glutathione in the case of salt stress, which may account for the salt-tolerant trait of the plant.

l-gulonolactone oxidase-like (K7U1M0) was downregulated in Chang7-2 (0.32-fold) but upregulated in Zheng58 (2.57-fold) after 0.5 h of salt treatment. l-gulonolactone oxidase is the key enzyme in the synthesis of ascorbic acid [67]. Ascorbic acid has a strong reducing capability; it is, like glutathione, an important antioxidant. We observed increased H_2_O_2_ content in response to salt treatment in Zheng58 roots (Figure 1c), which required more antioxidant to remove H_2_O_2_, consistent with the phosphoproteomics results. The abundance of ascorbate peroxidase was reported to be upregulated in a salt-tolerant maize line [54], suggesting that enhanced synthesis of glutathione and ascorbic acid for scavenging ROS is a common strategy for salt-tolerant plants.

#### 3.2.3. Glutamate Metabolism

Glutamate decarboxylase (B4F972) promotes the decarboxylation of glutamate to produce γ-aminobutyric acid (GABA) and CO_2_ [68]. Salt treatment (0.5 h) induced a significant decrease in phosphorylation of the protein in Zheng58, though it slightly recovered at 2 h. The decomposition of glutamate was inhibited when the phosphorylation of glutamate decarboxylase was decreased, leading to the accumulation of glutamate. Glutamate is the major substrate for the synthesis of proline under stress conditions [69]. Increased proline content is an adaptive response of plants to salt stress [12,70,71]. In addition, glutamate synthase (A0A096RAH5), which is involved in glutamate synthesis, was upregulated in Zheng58 but unchanged in Chang7-2. These results indicate that Zheng58 could maintain a higher level of glutamate under salt stress conditions, which can be used for the synthesis of proline, and this may contribute to its salt tolerance trait. A similar result was found in the comparative proteomics study between other maize lines [54].

#### 3.2.4. Transport-Related Proteins

Under salt stress, sodium and potassium ions are selectively absorbed by plants; potassium instead of sodium is the preferred metal ion for absorption [5]. A previous study reported that salt-tolerant plants have higher concentrations of potassium in the extended tissues [72]. Potassium channel AKT1 (K7V3Z4) enables the transmembrane transfer of potassium ions by a voltage-gated channel and plays vital roles in K^+^ uptake [73]. The phosphorylation of potassium channel AKT1 was upregulated in Zheng58 (2.06-fold at 0.5 h treatment), which enhanced its ability to transport potassium, resulting in its salt tolerance. In Chang7-2, the phosphorylation of this protein remained unchanged (a 1.28-fold change at 0.5 h treatment). Also, the phosphorylation of high-affinity potassium transporter (W5U5W2) was increased in Zheng58 under salt stress, hinting that it is closely related to the salt tolerance of Zheng58.

Sodium/hydrogen exchanger (A0A096Q7K1) has sodium/proton antiporter activity and pumps sodium ion from the cytoplasm to the vacuole, which not only reduces the damage that Na^+^ exerts on enzymes and the membrane system, but also alleviates osmotic stress imposed by an elevated Na^+^ concentration [7]. Maintaining a lower Na^+^/K^+^ ratio in the cytoplasm by pumping Na^+^ into the vacuole is an important mechanism for plants to cope with salt stress. Two V-type proton ATPases were found to be upregulated after salt treatment in sugar beet to enhance the Na^+^ compartmentalization into vacuoles and reduce Na^+^ accumulation in the cytosol [74]. The phosphorylation level of the sodium/hydrogen exchanger remained unchanged in Chang7-2 but was significantly increased in Zheng58 at 0.5 h treatment (Table 1), evidencing that Zheng58 was more salt-tolerant.

Calcium is a signaling molecule ubiquitously existing in plants and is considered to play important roles in response to salt stress. Salt stress causes an increase in intracellular calcium concentration, and this signal is sensed and passed down by related proteins and eventually results in activation of target kinase and transcription factors and the expression of downstream regulating genes to initiate the salt-resistance mechanism [75,76,77]. Calcium/proton exchanger CAX1-like protein (B4F910) translocates Ca^2+^ and other metal ions into vacuoles using the proton gradient formed by H^+^-ATPase and H^+^-pyrophosphatase [78]. Phosphorylation of this protein was decreased at 2 h of salt treatment in Zheng58 but remained unchanged in Chang7-2 (Table 1), so more Ca^2+^ can be accumulated in the cytoplasm of Zheng58 under saline conditions, which facilitated the quick initiation of signaling transduction to resist salt stress.

### 3.3. Functional Analysis of DRPPs in Shoots of Zheng58 and Chang7-2 under Salt Stress

#### 3.3.1. Photosynthesis-Related Proteins

Many photosynthesis-associated proteins have been reported to be accumulated in *Sorghum bicolor* in response to salt stress [28]. In our study, photosystem I reaction center subunit IV A (B6TH55) and Photosystem II reaction center protein H (P24993) were both upregulated in phosphorylation in Zheng58 (2.13-fold and 2.44-fold, respectively) but remained unchanged in Chang7-2 (Table 2). This indicated that photosynthesis was enhanced in Zheng58 under short-term salt stress, suggesting that salt stress could stimulate photosynthesis in salt-tolerant plants to supply more energy for the plants to resist salt stress.

#### 3.3.2. Proteins Involved in Carbon Metabolism

Carbon metabolism is greatly affected in plants under saline conditions [28,74]. Phosphoenolpyruvate carboxylase 1 (PEP1) (Q43267) catalyzes the reaction between phosphoenolpyruvate and carbon dioxide to form oxaloacetate, which is involved in the TCA cycle to provide energy for plants [79]. The phosphorylation of PEP1 was decreased in both inbred lines at 0.5 h of salt treatment and then significantly increased at 2 h; the increase was greater in Zheng58 than in Chang7-2 (6.46-fold versus 4.72-fold); thus, in Zheng58, carbon metabolism was enhanced to produce more energy for salt stress relief. Phosphoenolpyruvate carboxykinase (Q9SLZ0) functions oppositely to PEP1: it converts oxaloacetate to phosphoenolpyruvate and carbon dioxide [80]. The phosphorylation of phosphoenolpyruvate carboxykinase was significantly downregulated in Zheng58 shoots after salt treatment (0.08-fold and 0.27-fold at 0.5 and 2 h treatment, respectively), leading to the accumulation of oxaloacetate that eventually got into the TCA cycle. UDP-glucose 6-dehydrogenase (A0A096T909) is responsible for turning UDP-glucose into UDP-glucuronic acid. UDP-glucuronic acid is one of the most important compounds for polysaccharide synthesis [81]. Studies have shown that polysaccharide acts as an osmotic regulator to protect plants from stress caused by high salt conditions [82,83]. The phosphorylation of UDP-glucose 6-dehydrogenase was downregulated after 0.5 h of salt stress and slightly recovered after 2 h in Chang7-2; it maintained significant downregulation in Zheng58 within the two hours of salt treatment (Table 2). These results suggest that short-term salt treatment did not induce but rather reduced polysaccharide synthesis. Thus, we proposed that, in the early stage of salt stress, plants produced more energy through the TCA cycle and reduced energy consumption in the synthesis of polysaccharide, thus providing more energy for salt relief processes, such as ion transport and synthesis of substances for cell growth.

#### 3.3.3. Glutathione Metabolism

Two glutathione transferases, glutathione S-transferase (B4FTF8) and glutathione S-transferase 6 (B6T7H0), were identified as DRPPs in shoots. These two glutathione transferases were both increased in phosphorylation in Zheng58 at 0.5 h of salt treatment and slightly decreased at 2 h, while they both remain unchanged in Chang7-2 (Table 2). The abundance of glutathione S-transferase was also upregulated under salt stress in wheat [31]. Glutathione transferases catalyze the binding reaction of glutathione and various electrophilic compounds [66]. We suspected that the phosphorylation of glutathione transferases helped to eliminate some toxic substances that were produced in the shoot under salt stress.

#### 3.3.4. Plant Hormone Signal Transduction

Abscisic acid-insensitive 5-like protein (B4F831) is a bZIP transcription factor involved in the ABA signaling pathway [84]. ABA is a stress-signaling molecule that plays pivotal roles in stressful environments, such as drought, cold, and salt [85,86,87]. It has been documented that BnaABF2, a bZIP transcription factor from rapeseed, enhanced the salt tolerance in transgenic Arabidopsis [88]. The phosphorylation of B4F831 was upregulated initially and slightly decreased afterwards in Chang7-2; however, in Zheng58, it showed sustained significant upregulation. The large increase of B4F831 in Zheng58 may contribute to its salt tolerance. The increased phosphorylation of this protein enhanced the downstream expression of stress-related genes. In addition, an RNA-binding protein involved in signal transduction, Ninja-family protein 6 (B6TNQ7), was identified as a DRPP and was also upregulated in Zheng58 but showed no significant change in Chang7-2 after NaCl treatment.

## 4. Materials and Methods

### 4.1. Plant Materials and NaCl Treatment

Maize recombinant inbred lines (RILs) Zheng58 and Chang7-2 were kindly provided by Prof. Yan He from the China Agricultural University. Seeds were germinated in wet sand at room temperature (about 22–25 °C) with 40−60% humidity. Seven-day-old seedlings were uprooted from sand, and their roots were rinsed with distilled water and transferred to pots filled with Hoagland nutrient solution (Macronutrients: K_2_SO_4_, Ca(NO_3_)_2_·4H_2_O, KH_2_PO_4_, MgSO_4_·7H_2_O, Fe-EDTA, KCl; micronutrients: H_3_BO_3_, MnSO_4_·H_2_O, ZnSO_4_·7H_2_O, CuSO4·5H_2_O, (NH_4_)_6_Mo_7_O_24_·4H_2_O). The nutrient solution was replaced every other day. Seedlings at the trifoliate stage were treated with 100 mM NaCl [54,55,56] for 0 h, 0.5 h, and 2 h, and the roots and shoots were collected afterwards. Parts of the plant were used to measure physiological parameters immediately after treatment, and the remaining samples were frozen in liquid nitrogen and stored at −80 °C for further analysis.

### 4.2. Physiological Parameter Measurements

Roots and shoots were dried at 80 °C to a constant weight for 24 h. The dry weights of the samples were measured. The dried roots and shoots were incinerated in a muffle furnace at 300 °C for 3 h and 575 °C for 6 h. The ashes were dissolved in 10 cm^3^ 5% nitric acid and diluted with 5% nitric acid accordingly. The concentrations of macroelements (Na, K, Ca, and Mg) and microelements (Cu, Fe, Mn, and Zn) in the digested solution were determined using a 4100-MP ICP-OES (Agilent, Santa Clara, CA, USA) [89]. Three biological replicates were tested.

Proline contents in fresh plant tissues were measured using the AccQ-Tag derivatization kit (Waters, Milford, MA, USA) with LC-MS, and three biological replicates were examined. In brief, 20 mg lyophilized plant tissue powder was extracted with 1 cm^3^ water under sonication. The extract was derivatized with the Waters AccQ-Tag amino acid derivatization kit and analyzed with LC-MS [90].

H_2_O_2_ contents were measured using the Amplex Red Hydrogen Peroxide/Peroxidase Assay Kit (Thermo Scientific, Waltham, MA, USA) according to manufacturer’s instructions. Each sample was subjected to three biological replicates and two technical replicates.

### 4.3. Protein Extraction and Digestion

Protein extraction was adopted from a published method with a slight modification [91]. Two biological replicates and two technical replicates were measured. Two grams of frozen plant samples were ground to a fine powder with a mortar in liquid nitrogen. Extraction buffer (6 cm^3^) (500 mM Tris–HCl, 500 mM EDTA, 700 mM sucrose, 100 mM KCl, pH 8.0, 1% protease inhibitor cocktail, 1% phospho-STOP) was added, and the powder was ground for 10 min. Then, 6 cm^3^ Tris saturated phenol was added, and the powder was ground for another 10 min. The phenol layer was collected after centrifugation and the protein was precipitated with 0.1 M ammonia acetate in methanol. The protein precipitate was washed with cold acetone and dried under a vented hood.

The protein was dissolved in 7 M urea and 2 M thiourea. The concentration of the protein solution was measured by the Bradford assay. The protein was digested with trypsin using a modified FASP method [92]. In short, the protein solution was loaded onto an ultrafiltration device (30 kDa, 500 mm^3^, Sartorius, Gottingen, Germany), washed with 50 mM NH_4_HCO_3_, reduced with 200 mM DTT at 56 °C, and alkylated with 200 mM iodoacetamide in the dark. The protein was digested with trypsin at 37 °C overnight (enzyme: protein ratio = 1:50). Parts of the digested peptides were diluted with 0.1% FA for nano LC-MS analysis. The remaining peptides were lyophilized for phosphopeptide enrichment.

### 4.4. Phosphopeptide Enrichment

Phosphopeptides were enriched using TiO_2_-tips (Thermo Scientific, Waltham, MA, USA) according to the manufacturer’s protocol. The phosphopeptides were reconstituted in 0.1% FA for nano LC-MS analysis.

### 4.5. Mass Spectrometry Analysis

Nano LC-MS analysis was performed on a Waters nano-Acquity nano HPLC (Waters, Milford, MA, USA) coupled with a Thermo Q-Exactive high resolution mass spectrometer (Thermo Scientific, Waltham, MA, USA). A peptide solution (7 mm^3^) was loaded onto a trap column (AcclaimPepMap100, 75 μm × 2 mm, Thermo Scientific, Waltham, MA, USA). The analytical column was a 100 μm I.D. fused silica capillary filled with 20 cm of C_18_ stationary phase (Aqua C_18_, 3 μm, 125 Å, Phenomenex, Los Angeles, CA, USA). A 125 min gradient was used to elute the peptides. Mobile phase A was 0.1% FA in water, B was 0.1% FA in acetonitrile, and the flow rate was 400 nL/min. The nano ESI spray voltage was 2.0 kV and a full mass scan in the range of m/z 300–1800 was obtained with a resolution of 70,000 at m/z 200. The 10 most intensive peptide signals from the full scan were selected for an MS/MS scan with a resolution of 17,500 at m/z 200. The dynamic exclusion time was 20 s.

### 4.6. Protein Identification and Quantification

Raw data were imported into Progenesis QI for Proteomics (build 2.0, Nonlinear Dynamics, Newcastle, UK) for peak alignment and peak picking. The obtained MGF files were submitted to the Mascot search engine (version 2.5.0, Matrix Science, London, UK) and searched against the Uniprot maize database (58,418 sequences, 2015-12, https://www.uniprot.org/). Trypsin was selected as the specific enzyme with a maximum of two missed cleavages. Fixed modification was carbamidomethyl (C). Oxidation (M) and phosphorylations (S, T, and Y) were set as variable modifications. The MS mass tolerance was 10 ppm and the MS/MS mass tolerance was 0.02 Da. A decoy database was used, and peptides were filtered at 1% FDR. Peptides with a mascot score higher than 29 were selected for further analysis. The mass spectrometric data were deposited at Integrated Proteome Resources with the project No. IPX0001523000 (https://www.iprox.org).

### 4.7. Bioinformatics Analysis

The GO enrichment analysis and MFUZZ analysis were conducted with the help of the Wukong platform (http://www.omicsolution.org/wu-kong-beta-linux/main/). Protein−protein interactions were analyzed by STRING 10.5 (http://string-db.org/) [62] using the DRPPs as input.

### 4.8. Statistical Analysis

Hydrogen peroxide and proline contents were measured in six and three biological replicates, respectively, and metal ion contents were determined in three biological replicates. Statistical analysis was performed using Student’s *t*-test in Excel. Significant differences are indicated by an asterisk. One asterisk indicates a significant difference at the 0.05 level and two asterisks indicate a difference at the 0.01 level. Four biological replicates were performed in the phosphoproteomic experiment, and ANOVA was used to calculate the *p*-values. Proteins were considered to have significant changes in abundance when a *p*-value of <0.05 was reached, with a fold change of >2. Hierarchical cluster analysis of DRPPs was performed using cluster 3.0 (https://cluster.updatestar.com/en/edit) and Treeview software (http://jtreeview.sourceforge.net/). PCA analysis was conducted using SIMCA 13.0 software (Umetrics, Umeå, Sweden).

## 5. Conclusions

In conclusion, we explored the mechanism of salt tolerance in maize by comparing differentially regulated phosphoproteins in the maize inbred lines Chang7-2 and Zheng58 under short-term salt stress. Zheng58 had higher Na^+^ content in roots, lower Na^+^ content in shoots, and a lower Na^+^/K^+^ ratio after salt treatment, which were crucial for maintaining salt tolerance. The H_2_O_2_ content in the roots of Zheng58 was significantly increased after salt stress, and the accumulation of H_2_O_2_ acted as a stimulator to enhance Zheng 58′s salt tolerance. Proline, an osmotic regulating compound, was increased in both inbred lines after 0.5 h of salt treatment, and the increase was greater in Zheng58 than in Chang7-2. The content of proline in shoots changed to the opposite direction. These results indicate that, under short-term salt stress, proline that is already in the shoots is recruited to the roots to cope with salt stress. Moreover, Zheng58 was able to transport proline more efficiently from shoots to roots to cope with salt stress. Several phosphoproteins related to salt tolerance were found to be significantly regulated in Zheng58. In roots, 6-phosphogluconate dehydrogenase 2, pyruvate dehydrogenase, and phosphoenolpyruvate carboxykinase provided NADPH and energy for the plants to resist salt stress. Glutathione S-transferase 3 and l-gulonolactone oxidase-like were related to glutathione and ascorbic acid metabolism, which offered antioxidants to remove ROS, such as H_2_O_2_. The phosphorylation changes in glutamate decarboxylase and glutamate synthase in Zheng58 under NaCl treatment were favorable for the accumulation of glutamate, which acts as a major substrate of proline synthesis under stress conditions. Potassium channel AKT1, High-affinity potassium transporter, and sodium/hydrogen exchanger were found to be related to potassium and sodium transportation. The upregulation of these proteins in Zheng58 under salt stress resulted in a lower Na^+^ content in shoots and a lower Na^+^/K^+^ ratio in both roots and shoots in Zheng58. Calcium/proton exchanger CAX1-like protein was found to be related to calcium ions transportation. The downregulation of this protein in Zheng58 after salt treatment resulted in accumulation of Ca^2+^ in the cytoplasm to initiate the salt signal transduction pathway. In shoots, photosystem I reaction center subunit IV A and photosystem II reaction center protein H, which are involved in photosynthesis, supplied more energy to Zheng58. PEP1 and phosphoenolpyruvate carboxykinase, which are involved in carbon metabolism, promote the degradation of carbohydrates to produce more energy. The downregulation of UDP-glucose 6-dehydrogenase was helpful to save more energy for salt relief processes. Two glutathione transferases that are involved in eliminating some toxic substances produced during salt stress were found to be upregulated in Zheng58. Abscisic acid-insensitive 5-like protein, which is involved in the ABA signaling pathway, enhanced the expression of stress-related genes in Zheng58 under salt stress.

## Figures and Tables

**Table 1:** Details of differentially regulated phosphoproteins related to salt tolerance in Zheng58 roots.

Accession	Annotation	*p*-Value	C-0.5h	C-2h	Z-0.5h	Z-2h
			fc ^1^	fc	fc ^2^	fc
**Carbon Metabolism**						
B4FSV6	6-phosphogluconate dehydrogenase 2	0.0095	0.37	0.31	1.88	2.05
B4FGJ4	Pyruvate dehydrogenase	1.1 × 10^−6^	0.08	0.06	3.60	2.02
C0P3W9	phosphoenolpyruvate carboxykinase	0.0124	0.38	0.38	2.16	1.58
**Glutathione and Ascorbic Acid Metabolism**						
B4FK84	Glutathione S-transferase 3	0.0427	0.47	1.59	0.04	0.53
K7U1M0	l-gulonolactone oxidase-like	0.0054	0.32	0.19	2.57	1.37
**Glutamate Metabolism**						
B4F972	Glutamate decarboxylase	0.0203	-	-	0.30	0.80
A0A096RAH5	glutamate synthase	0.0002	1.01	0.79	2.00	1.53
**Transport-Related Proteins**						
K7V3Z4	Potassium channel AKT1	0.0092	1.28	0.99	2.06	1.39
W5U5W2	high-affinity potassium transporter	0.0095	1.07	0.95	2.49	0.78
A0A096Q7K1	Sodium/hydrogen exchanger	4.29 × 10^−6^	1.55	1.64	2.13	0.71
B4F910	Calcium/proton exchanger CAX1-like protein	1.01 × 10^−5^	1.17	0.99	1.16	0.35

^1^ C-0.5h fc indicates a fold change at 0.5 h of salt treatment compared with the control group in Chang7-2. ^2^ Z-0.5h fc indicates a fold change at 0.5 h of salt treatment compared with the control group in Zheng58.

**Table 2:** Details of differentially regulated phosphoproteins related to salt tolerance in Zheng58 shoots.

Accession	Annotation	*p*-Value	C-0.5h	C-2h	Z-0.5h	Z-2h
			fc	fc	fc	fc
**Photosynthesis-Related Proteins**						
B6TH55	Photosystem I reaction center subunit IV A	2.45 × 10^−8^	1.56	1.21	2.13	1.60
P24993	Photosystem II reaction center protein H	8.67 × 10^−8^	1.32	1.16	0.53	2.44
**Carbon Metabolism**						
Q43267	phosphoenolpyruvate carboxylase 1 (PEP1)	8.42 × 10^−9^	0.51	4.72	0.39	6.46
Q9SLZ0	Phosphoenolpyruvate carboxykinase	1.65 × 10^−11^	0.26	0.90	0.08	0.27
A0A096T909	UDP-glucose 6-dehydrogenase	8.58 × 10^−12^	0.05	0.77	0.04	0.26
**Glutamate Metabolism**						
B4FTF8	glutathione S-transferase	1.12 × 10^−7^	1.18	1.40	2.05	1.74
B6T7H0	glutathione S-transferase 6	9.47 × 10^−7^	1.10	0.75	2.07	1.25
**Plant Hormone Signal Transduction**						
B4F831	Abscisic acid-insensitive 5-like protein	9.29 × 10^−9^	2.05	1.52	2.36	2.50
B6TNQ7	Ninja-family protein 6	6.63 × 10^−9^	1.93	1.88	2.20	2.53

## Supplementary Materials

Supplementary Materials can be found at https://www.mdpi.com/1422-0067/20/8/1886/s1.

## Abbreviations

bZIP	Basic region-leucine zipper
DRPPs	Differential regulated phosphoproteins
FDR	false discovery rate
GABA	γ-aminobutyric acid
GO	Gene ontology
ICP-OES	Inductively Coupled Plasma Optical Emission Spectrometry
PCA	Principal component analysis
PEP1	Phosphoenolpyruvate carboxylase 1
PIP	plasma membrane intrinsic proteins
PSMs	Peptide spectrum matches
RILs	Recombinant inbred lines
ROS	Reactive oxygen species
SOS	Salt Overly Sensitive
STRING	Search Tool for the Retrieval of Interacting Genes
TCA	Tricarboxylic acid
